# Pathobiology of Hodgkin Lymphoma

**DOI:** 10.4084/MJHID.2014.040

**Published:** 2014-06-05

**Authors:** Claudio Agostinelli, Stefano Pileri

**Affiliations:** Section of Haematopathology, Department of Experimental, Diagnostic and Specialty Medicine – DIMES, University of Bologna, Italy.

## Abstract

Hodgkin’s lymphoma is a lymphoid tumour that represents about 1% of all de novo neoplasms occurring every year worldwide. Its diagnosis is based on the identification of characteristic neoplastic cells within an inflammatory milieu. Molecular studies have shown that most, if not all cases, belong to the same clonal population, which is derived from peripheral B-cells. The relevance of Epstein-Barr virus infection at least in a proportion of patients was also demonstrated. The REAL/WHO classification recognizes a basic distinction between nodular lymphocyte predominance HL (NLPHL) and classic HL (CHL), reflecting the differences in clinical presentation, behavior, morphology, phenotype, molecular features as well as in the composition of their cellular background. CHL has been classified into four subtypes: lymphocyte rich, nodular sclerosing, mixed cellularity and lymphocyte depleted. Despite its well known histological and clinical features, Hodgkin’s lymphoma (HL) has recently been the object of intense research activity, leading to a better understanding of its phenotype, molecular characteristics and possible mechanisms of lymphomagenesis.

## Introduction

In the first half of 19^th^ century, Sir Thomas Hodgkin provided the first macroscopic description of the process, that Samuel Wilkins named Hodgkin Disease (HD) in a paper entitled “On some morbid appearances of the absorbent glands and spleen”. In 1898 and 1902, Carl Sternberg and Dorothy Reed independently described the typical “diagnostic” cells. In 1956, Smetana and Cohen identified a histopathological variant of granulomatous HD, which was characterized by sclerotic changes and better prognosis. This variant was termed “nodular sclerosis HD” in the classification proposed by Lukes, Butler, and Hicks in 1964, and its simplified form, produced by the Rye conference, has been used routinely until 1994 when it was replaced with the revised European–American lymphoma (REAL) classification.^1^ In the latter the Hodgkin lymphoma (HL) was listed in and subdivided into two main types: nodular lymphocyte predominant (NLPHL) and classical HL (CHL), in the light of morphological, phenotypic, genotypic, and clinical findings. CHL included the following subtypes: nodular sclerosis (NS-CHL), mixed cellularity (MC-CHL), lymphocyte-depleted (LD-CHL) and the lymphocyte rich CHL (LR-CHL). This approach has finally been adopted by the World Health Organisation (WHO) scheme in 2001^2^ and confirmed in the WHO classification 2008 (Table 1).^3^

## Nodular Lymphocyte Predominant Hodgkin Lymphoma

NLPHL represents 4–5% of all HL cases. Most patients present with localized disease (stage I or II), usually affecting single cervical, axillary, or inguinal nodes; splenic, bone marrow and mediastinal involvement is rare.^4^ The tumour has a very indolent course, with prolonged disease free intervals, despite a high rate of late relapses, which usually respond well to treatment.^4^ Progression to a diffuse large B cell lymphoma (DLBCL) has been reported in 3–5% of case, which has a more favourable outcome than de novo large B cell lymphomas.^4^

### Morphology

**Figure 1:**
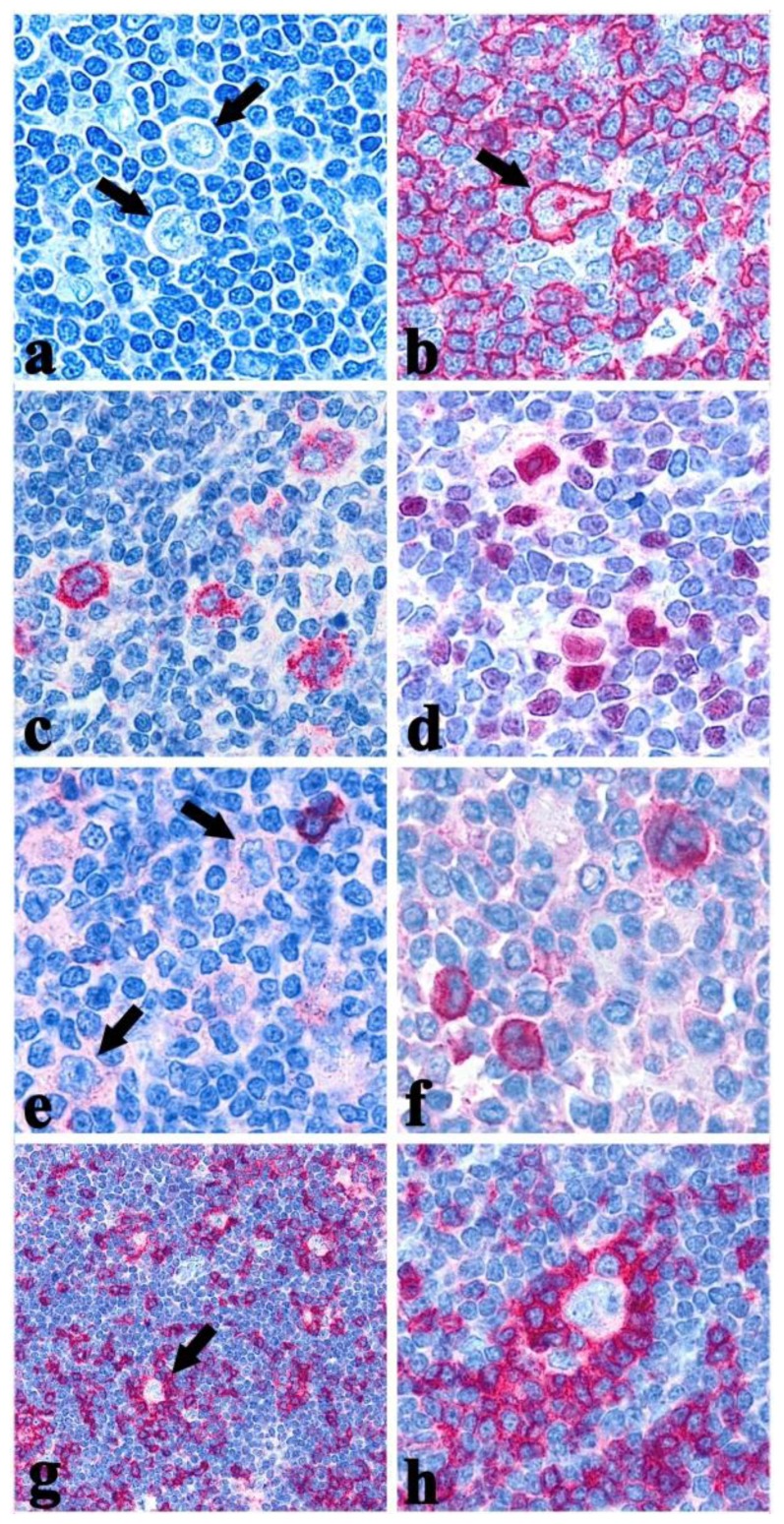
**a)** Lymphocyte Predominant (LP) cells in NLPHL with typical popcorn morphology indicated by the two arrows (×400 Giemsa stain); **b)** LP cell expressing CD20 (arrow) (×400); **c)** LP cells EMA^+^ (×400); **d)** BCL6 positivity in LP cell (×400); **e)** CD30 negativity in LP cells; note the mononuclear non neoplastic CD30+ blasts (×400); **f)** LP cells IgD^+^**; g)** the arrow highlight a rosette of CD3^+^ small T lymphocytes around LP cells (×200)**; h)** rosettes of PD1^+^ small lymphocytes around LP cells (×400).

NLPHL differs greatly from the classical type in terms of morphology and the only feature shared with CHL is the paucity of the neoplastic population which consists of large elements, formerly called L&H (lymphocytic/histiocytic) or popcorn cells and now designated as LP (lymphocyte predominant) cells (Figure 1, a) in the 4^th^ edition of the WHO Classification.^4^ The lymph node architecture is totally or partially effaced by a nodular or a nodular and diffuse infiltrate consisting of small lymphocyte, histiocytes, epitheliod elements admixed with LP cells.^5^ The latter show nuclei with a polylobular profile, finely dispersed chromatin and multiple, basophilic small nucleoli resembling those of centroblasts, which are often adjacent to the nuclear membrane.^5^ Occasionally, LP may display the features of Hodgkin Reed-Sternberg (HRS) cells and in this settings immunophenotyping plays a pivotal role for the differential diagnosis between NLPHL and LR-CHL. Exceptionally, co-occurrence of clonally related NLPHL and CHL has been reported.^6^,^7^

In 2003, Fan et al. proposed a sub-classification of LP-HL into 6 categories based on the immunoarchitectural pattern (Table 2): the diffuse (TCRBCL-like) one (pattern E) mimics a T-cell/histiocyte rich large B cell lymphoma (THCRBCL)().^8^ According to the current WHO classification, at least a partial nodular pattern is required for a diagnosis of NLPHL; a purely diffuse pattern would be classified as TCRBCL.^4^ Occasionally, NLPHL can be confused with a peculiar form of follicular hyperplasia with progressive transformation of germinal centres (PTGC).^9^ These lesions occur in children and young adults and may precede, concur with, or follow NLPHL. It is uncertain weather PTGCs are preneoplastic, nevertheless they seem to be associated with a slightly higher risk of developing NLPHL than the average population.^9^ On morphological grounds, PTGCs are two to three times larger than reactive follicles and predominantly consist of small lymphocytes, mainly mantle cells, intermingled with some centroblasts and follicular dendritic cells (FDCs).^9^ PTGCs can be differentiated from NLPHL because of the lack of LP elements.^9^

### Immunophenotype

The neoplastic cells have a characteristic B cell profile: in particular, they are CD45^+^, CD20^+^ (Figure 1, b), CD79a^+^, CD22^+^, PU.1^+/−^, Oct-2^+^, BOB.1^+^, J-chain^+/−.4,10,11^ LP are positive for epithelial membrane antigen (EMA) (Figure 1, c) and lack CD30 (Figure 1, e), CD15 and LSP1.^4^ Interestingly, a certain number of extrafollicular reactive mononucleated blasts (smaller than popcorn cells) are detected by the anti-CD30 antibodies but the latter are unrelated to the neoplastic population (Figure 1, e).^4^ LP cells are often IRF4^+^ and regularly express the germinal center marker BCL6 (Figure 1, d), being CD10^−;4^ in contrast to CHL cells, they maintain the expression of the transcription factor Oct2 and its coactivator BOB.1.^10^ Heavy and light chains of the immunoglobulins are frequently expressed and, in particular, IgD positivity identifies a sub-group of cases (9–27%) with peculiar epidemiological, phenotypical (IgD^+^, CD38^+^, CD27^−^, IgM^−^) and clinical features (Figure 1, f).^12^

The reactive background in NLPHL is characterized by a large spherical follicular dendritic cell (FDC) meshwork CD21^+^/CD35^+^, within the nodules, which are filled with small lymphocytes that mainly consist of B cells and follicular helper T elements (CD3^+^/CD4^+^/CD57^+^/PD1^+^) forming rosettes around LP cells (figure 1, g, h). The progression to a diffuse form, however, is characterised by the increase of T-lymphocytes that can finally predominate over B-cells.^4^,^13^ The rosetting of small lymphocytes CD4^+^/CD57^+^/PD1^+^ around typical LP cells is indeed useful for the differential diagnosis with PTGC, LR-CHL, and TCRBCL.^13^ In addition, staining for LSP1, PU1 and IgD has to be considered.^11^

### Genetics

Molecular studies, based on the single cell polymerase chain reaction (PCR) demonstrated that LP cells have clonally rearranged immunoglobulin *(IG)* gene with high load of somatic mutations in the variable region of *IG* heavy chains genes.^14^ These rearrangements are usually functional and *IG* mRNA transcripts and protein are detectable in LP cells. Ongoing mutations are detected in about half of NLPHL cases:^14^,^15^ this finding identifies mutating germinal centre cells as the precursors of the neoplastic elements. In addition, an aberrant somatic hypermutation process targeting PAX5, RHOH/TTF, PIM1, and MYC has been recorded in 80% of NLPHL cases, further supporting the germinal center derivation.^16^ In line with these data, studies of gene expression profile (GEP) carried on isolated neoplastic cells indicate that LP cells possibly originate from germinal center B cells at the transition to memory B cells. Recently, using the same approach, Hartmann et al performed GEP of microdissected tumour cells of NLPHL, THRLBCL-like NLPHL and THRLBCL and interestingly in unsupervised analyses, the examples of the three types of lymphoma did not form separate clusters.^17^ Moreover, even in supervised analyses, very few consistently differentially expressed transcripts were found, and for these genes the extent of differential expression was only moderate. The authors concluded that there are no clear and consistent differences in the gene expression of the tumour cells of NLPHL, THRLBCL-like NLPHL and THRLBCL. Furthermore, the characterization of the tumour microenvironment for infiltrating T cells and histiocytes revealed significant differences in the cellular composition between typical NLPHL and THRLBCL cases, but THRLBCL-like NLPHL presented a pattern more related to THRLBCL than NLPHL. In conclusion, the authors propose that NLPHL and THRLBCL may represent a spectrum of the same disease and that the different clinical behaviour of these lymphomas may be strongly influenced by differences in the lymphoma microenvironment, possibly related to the immune status of the patient at the time of diagnosis.

Finally, Epstein-Barr virus (EBV) has never been detected in the LP cells, in contrast to the neoplastic component of CHL.^4^

## Classical Hodgkin Lymphoma

**Figure 2:**
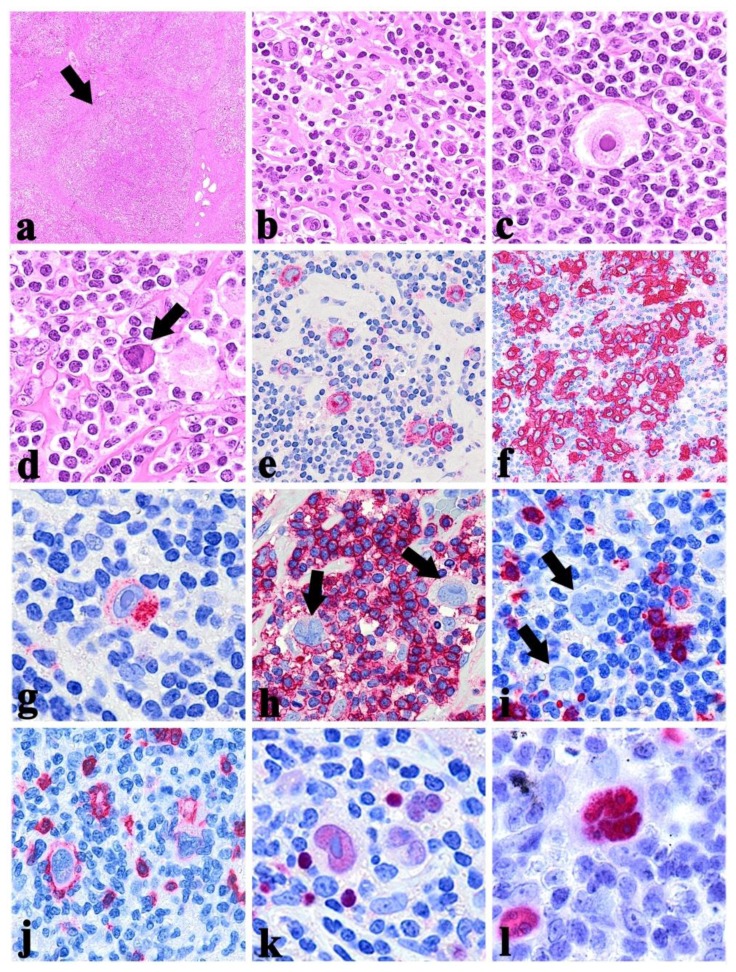
**a)** Nodular growth pattern in a typical case of NS-CHL; one of the nodules is indicated by the arrow (×20, Hematoxylin-Eosin); **b)** Hodgkin Reed-Sternberg (HRS) and Hodgkin (H) cells in a CHL (×400, Hematoxylin-Eosin); **c)** lacunar cell in a NS-CHL case (×400, Hematoxylin-Eosin); **d)** mummified cell showed by arrow in a MC-CHL case (×400, Hematoxylin-Eosin); **e)** HRS and H cells CD30^+^; note the typical membranous and dot-like (in Golgi area) staining pattern (×400); **f)** HRS and H cells CD30^+^ in a case of NS-CHL syncytial variant (×200) ; **g)** H cell showing dot-like CD15 positivity (×400); **h)** the arrows indicate two H cells CD45^−^ in a background of CD45^+^ reactive lymphocytes (×400); **i)** H cells CD20^−^ highlighted by the arrows (×400); **j)** irregular expression of CD20 in HRS cells (×400); **k)** H cell PAX5/BSAP^+^; note the weaker expression in comparison with small reactive B lymphocytes (×400); **l)** EBV viral integration in the genome of HRS cells, revealed by in situ hybridization reaction using anti-EBER1/2 probes (×400).

The diagnosis of classical Hodgkin lymphoma is based on the identification of characteristic multinucleated giant cells – termed Hodgkin Reed-Sternberg (HRS) or diagnostic cells – within an inflammatory milieu.^18^ These cells measure 20–60 μm in diameter and display a large rim of cytoplasm and at least two nuclei with acidophilic or amphophilic nucleoli, covering more than 50% of the nuclear area (Figure 2, b).^18^ The tumoral population also includes a variable number of mononuclear elements – Hodgkin’s (H) cells – showing similar cytological features to HRS cells (Figure 2, b). Although regarded as diagnostic, HRS cells are not exclusive of HL, since similar elements may be observed in reactive lesions (such as infectious mononucleosis), B- and T-cell lymphomas, carcinomas, melanomas or sarcomas.^18^ Thus, the presence of an appropriate cellular background – along with the results of immunophenotyping – is basic for the diagnosis. The reactive background, which can even represent 99% of the whole examined population, consists of small lymphocytes, histiocytes, epithelioid histiocytes, neutrophils, eosinophils, plasma cells, fibroblasts and vessels.^18^ HRS cells play a major role in the orchestration of the microenvironment milieu associated with HL. They can directly induce the recruitment of several immune cell types from the peripheral circulation and also trigger the local expansion of diverse cellular subsets. Recruitment of infiltrating immune cells is also stimulated by reactive cells themselves and particularly by macrophages and mast cells. Specifically, HRS cells synthesize and release many cytokines and chemokines as IL-5, IL-7, IL-8, IL-9, CCL-5, CCL-17, CCL-20, CCL-22 involved in the recruitment of granulocytes, lymphocyte, mast cells and macrophages.^19^ HRS also express a broad range of receptors including CD30, CD40, IL-7R, IL-9R, IL-13R, TACI and CCR5 able to detect growth and survival signals coming from the growth factor milieu.^19^ The final effect of this complex and dense network of signals mediated by direct cell contact and soluble mediator synthesis, is the delivery of pro-survival feedback to HRS cells.

Based on the characteristics of the reactive infiltrate and the morphology of HRS cells, four histological subtypes have been distinguished (Table 1): lymphocyte rich CHL (LR-CHL), nodular sclerosing CHL (NS-CHL), mixed cellularity CHL (MC-CHL), and lymphocyte depleted CHL (LD-CHL).^18^ The immunophenotype and the genetic features of HL and HRS cells are identical in these subtypes, but their clinical characteristics and association with EBV are different. How HRS cells develop is controversial and obscure; it has been postulated that these cells arise from mononucleated Hodgkin cells via endomitosis; however recently Rengstl et al. by tracking the cells and their progeny for multiple generations demonstrated that the fusion of daughter cells, termed re-fusion, plays an essential role in the formation of HRS cells in HL cell lines.^20^ Importantly, cell fusion events occur almost exclusively between cells sharing the same ancestor and visualization of the microtubule network in time-lapse microscopy experiments revealed a persistent connection between daughter cells in the majority of re-fusion events.^20^ This surprising finding supports the existence of a unique mechanism for the generation of multinuclear HRS cells that may have implications beyond HL, given that RS-like cells are frequently observed in several other lymphoproliferative diseases as well.

### Morphology

The lymph node architecture is effaced although remnants of normal follicles can be detected in some cases. Typical HRS and H cells represents a minority of the cellular infiltrate that consists of inflammatory elements. It should be underlined that some neoplastic cells appear “mummified” because of apoptotic changes (Figure 2, d).^18^ The composition of the reactive microenvironment and the structural alterations indeed vary in the histological subtypes MC-CHL, LD-CHL and LR-CHL.

#### • NS-CHL

It represents the most frequent subtype of classical HL in Italy and USA, where it corresponds to 75% of all CHL cases.^21^ The tumour is characterised by: sclerosis, lacunar cells and nodular pattern. Fibrotic phenomena correspond to the formation of broad collagen bands, which originate from a regularly thickened lymph node capsule and subdivide the lymphoid parenchyma into large nodules (Figure 2, a).^21^ The lacunar cells represent the cell variant of the HRS that tend to have more lobated nuclei, smaller nucleoli and a wide rim of clear or slightly acidophilic cytoplasm, very sensitive to formalin fixation (Figure 2, c). The latter in fact causes perinuclear condensation of the cytoplasm, which remains connected to the cell membrane via some narrow filaments, limiting empty “lacunar” cytoplasmic spaces.^21^ The nodules – which should be detected in at least part of the lymph node involved – can contain foci of necrosis and actually show a great variability in terms of inflammatory cell component (from lymphocyte predominace to lymphocyte depletion).

The British National Lymphoma Investigation (BNLI) Group subclassified nodular sclerosis into two grades (maintained also in WHO scheme).^21^ Grade II tumours seem to represent 15% – 25% of all nodular sclerosis cases and to run a more aggressive clinical course, a finding not confirmed by all studies. The term grade II is applied to cases showing one of the three following patterns:

1. more than 25% of the nodules have a cellular composition consistent with the pleomorphic or reticular subtype of LD-CHL;
2. more than 80% of the nodules display a fibrotic or fibro-histiocytic composition;
3. more than 25% of the nodules contain numerous large bizarre or anaplastic cells, in the absence of any depletion of the reactive small lymphoid component.

Two other variants of nodular sclerosis CHL are recognized: the so-called “cellular phase”, where there is a clear-cut tendency to nodule formation without overt collagen band deposition, and the “syncytial variant”.^21^ The latter is thought to represent 16% of all the NS-CHL cases and to run a more aggressive clinical course, as suggested by the occurrence of mediastinal bulky disease and stage III/IV in 88% of the patients. At light microscopy, it is characterised by large sheets of neoplastic cells, which may undergo central necrosis. In the past, similar cases have been diagnosed as non-Hodgkin’s lymphoma, metastatic melanoma, carcinoma or sarcoma or germ cell tumour.

#### • MC-CHL

About 15–25% of CHL cases belong to this group. The histological picture is characterised by a diffuse growth.^22^ The term MC-CHL reflects the cellular composition of the reactive milieu that consists of plasma cells, epithelioid histiocytes, eosinophils, and T-lymphocytes (CD3^+^/CD57^−^) forming rosettes around neoplastic elements.^22^ The latter correspond to HRS and H cells, that are rather numerous and easy to find, without lacunar or pop-corn variants. Two morphologic variants are reported: the interfollicular variant, that likely represents a partial lymph node involvement by CHL,^22^ and the epitheliod cell-rich variant, that shows prominent epithelioid cell reaction with granuloma formation.^22^

#### • LD-CHL

It is indeed rare, accounting for about 1% of HL cases, and is provided with the worst clinical behaviour and prognosis. In most instances, it is staged III–IV and displays B symptoms and bone-marrow involvement.^23^ Two subtypes of LD-CHL can be distinguished: fibrotic and reticular/sarcomatous.^23^ The former shows a low cellular density with small amounts of lymphocytes and prominent diffuse reticulin fibre formation, that includes variable number of HRS cells. The reticular/sarcomatous variant is instead characterised by the diffuse effacement of the normal lymph node by a huge amount of HRS cells, some of which appear “mummified”; small lymphocytes, plasma cells, histiocytes and granulocytes are scanty and foci of necrosis are usually found.^23^

#### • LR-CHL

LR-CHL accounts for about 6% of all HL cases. Morphologically, most cases show a vague nodularity, admixed histiocytes and absent neutrophils and eosinophils, thus closely resembling NLPHL.^24^ Furthermore, a proportion of the neoplastic cells can exhibit features of LP elements. Conversely to NLPHL, however, many lymphomatous cells have the morphologic features of classical HRS cells and the nodular structures frequently contain small germinal centres. Focal phenomena of sclerosis can sometimes be seen.^24^ The reactive component consists of abundant mantle B-cells and variable amounts of T-lymphocytes, which can produce rosettes around neoplastic elements.^24^ The clinical studies of the International Project on Lymphocyte Predominant Hodgkin’s Disease and the German Hodgkin’s Lymphoma Study Group^25^,^26^ have shown that patients with LR-CHL differ from those NS- or MC-CHL, since they are usually older than 50 display a higher incidence of stages I–II and sub-diafragmatic location, rarely have bulky disease, B symptoms, mediastinal or extranodal involvement, and experience more frequent late relapses, which are however provided with low aggressiveness. The clinical profile of lymphocyte-rich classical HL is thus closer to that of NLPHL, although its reveals a lower frequency of stages I–II and more common splenic infiltration.

### Phenotype

HRS and H cells express the CD30 molecule in more than 98% of CHL, although the intensity of the immunostining can vary from case to case and even within the same case.^18^ At immunohistochemical analysis, the antibodies against CD30 produce different types of positivity: membrane-bound, dot-like in the Golgi area (corresponding to the accumulation of the 90 kD proteic precursor), and diffuse (Figure 2, e, f).^18^ The first two patterns are exclusive of lymphoid elements with the exception of embryonic carcinoma, while the third one can occur in a variety of malignant tumours other than lymphomas, including pancreas carcinoma, naso-pharyngeal undifferentiated carcinoma, mesothelioma, and malignant melanoma.^27^ The CD30 molecule was already proposed as possible target for specific antibodies conjugated with I-131 and plant toxins and administered to patients with classical HL for therapeutic purposes: such antibodies have produced interesting results in cases refractory to conventional therapies, although severe hematotoxicity has at times been recorded.^28^ Recently, to enhance the antitumor activity of humanised CD30-directed therapy, the antitubulin agent monomethyl auristatin E (MMAE) was attached to a CD30-specific monoclonal antibody, producing the antibody-drug conjugate brentuximab vedotin (SGN-35), which is now proposed for most patients with relapsed or refractory CD30^+^ lymphomas.^29^

CD15 is detected in about 75–80% of cases of CHL. It is typically present in a membrane pattern with accentuation in the Golgi area and may be detected only in a minority of neoplastic cells (Figure 2, g).^18^ HRS and H cells generally lack CD45 and EMA expression (Figure 2, h).^18^ Despite their derivation from mature B cell, HRS and H cells are usually negative for the standard B cells markers (Figure 2, i).^18^ However, CD20 (Figure 2, j) and CD79a are found in 20–30% of the CHL samples.^30^ Positivity for one or more T-cell markers is detected in a minority of the cases and was recently associated with a worse prognosis.^18^,^31^ A characteristic finding is the absence of the transcription factor Oct-2, and its co-activator BOB.1, as well as of the transcription factor PU1.^10^ The B-cell nature of HRS is demonstrated in 98% of the cases only by PAX5/BSAP positivity (Figure 2, k).^32^ This molecule is physiologically expressed through all steps of B-cell differentiation with the exclusion of plasma cells,^32^ the immunostaining for the PAX5/BSAP is generally weaker in HRS and H cells than in normal B cells.^32^

Conversely to what observed in lymphocyte predominant HL, the elements of classical HL are generally BCL6^−^. In addition they are usually positive at high intensity for the plasma cell specific transcription factor IRF4, but plasmacell associated adhesion molecule CD138 is consistently negative.^18^

Immunophenotype largely contributes to differentiate CHL from anaplastic large cell lymphoma and from other B lineage lymphoma with overlapping clinical and morphological features of CHL, in particular the primary mediastinal large B-cell lymphoma (PMBL) and the B-cell lymphoma, unclassifiable, with features intermediate between diffuse large B-cell lymphoma and classical Hodgkin lymphoma.^33^ The latter is a new distinct entity (previously called grey zone lymphoma) introduced in the 4th edition of WHO classification.^33^ The clinical onset is often represented by a large anterior mediastinal mass which may involve the lung and may be associated to a vena cava syndrome. The disease frequently has a more aggressive clinical course and a poorer outcome than CHL and PMBL. The lymphoma is typically composed of pleomorphic neoplastic cells resembling lacunar cells and Hodgkin cells, with a sheet-like growth pattern; areas richer in centroblast-like cells are frequently observed. Similarly to DLBCL, the immunophenotype is characterized by the expression of CD45, of B-cell antigens CD20 and CD79a and transcription factors PAX5, BOB1 and OCT2, but is also associated to CHL markers CD30 and CD15 positivity.^33^ Sometimes a neoplasia resembling morphologically a PMBL but negative for CD20 can occur; in this instances a diagnosis of lymphoma with intermediate features between CHL and DLBCL can be made, if supported by CD15 and/or EBV positivity.^33^

### Genetics

The origin of RS cells of Hodgkin’s disease has long been unknown, mainly for its peculiar immunophenotypic profile. Molecular studies conducted by micromanipulation of single HRS cells from tissue sections and PCR analysis for rearranged *IG* genes have shown that in most, if not all cases, HRS and H cells belong to the same clonal population, which is derived from peripheral B-lymphocytes.^15^,^34^–^36^ Occasionally (2% of cases) a clonal rearrangement of the T-cell receptor genes was demonstrated.^18^ The rearranged *IG* genes harbour a high load of somatic hypermutations in the variable region of the heavy chain genes.^18^ Conversely to what seen in lymphocyte NLPHL, ongoing mutations are not usually detected in CHL, suggesting a derivation from a late germinal centre or post-germinal centre B-cell. Although HRS and H cells carry rearranged *IG* genes, they lack Ig synthesis;^18^ the latter features can be ascribed in 25% to the occurrence of mutations resulting in stop codons within originally functional rearrangements of the variable-region of the immunoglobulin heavy chain (*IgVH*) gene.^15^ Such mutations (defined as “crippling mutations”) are expected to occur in *IgVH* genes of germinal centre B-cells, but under physiologic conditions they should induce apoptosis of germinal centre cells, that result incapable of functional antibody expression.^15^ However, Marafioti et al. showed that crippling mutations are absent in 75% of classical HL cases, thus indicating that they cannot be responsible for the general absence of the *IG* transcripts.^36^ The latter event was then related to the downregulation of B-cell-specific transcription factors required for Ig expression, including *PU.1*, *Oct2*, and its co-activator *BOB.1*.^10^ This finding is characteristic of CHL and is not observed in normal B-cell subsets and B-cell non-Hodgkin lymphomas. It is not sustained by genomic imbalances or gene rearrangements, but seems to be related to epigenetic processes.^37^ Furthermore, HRS cells aberrantly express key transcription factors of other hematopoietic cell lineages as the T cell factor *Notch1* and the NK cell factor *ID2*, that antagonize the function of B cell genes.^38^–^40^ HRS and H cells also express multiple members (*BMI-1* and *EZH2*) of the polycomb group (PcG) family 1 and 2 complexes ).^41^–^43^ Some components of the PcG are present in normal B cells, but their co-expression is not seen in normal B cells. As *BMI-1* and *EZH2* can down regulate B cell genes, they may play a role in the characteristic down-regulation of the B cell program and the expression of markers of other lineages in HRS and H cells.^41^–^43^

Genetic instability is a characteristic feature of HRS and H cells that regularly exhibit numerical chromosome aberrations and the chromosome numbers are always in the hyperploid range. Chromosomal translocations affecting the *IG* loci are recurrent in CHL and involve partners as *BCL6*, *MYC*, *BCL3*, *RELB*, *REL* and *BCL2*.^44^,^45^ At least one among the *PIM1*, *PAX5*, *RhoH/TTF*, and *c-MYC* genes is involved by an aberrant somatic hypermutation process in 55% of classical HL cases, 2 or more being mutated in 30% of patients.^16^ Such rates are indeed lower than the ones recorded in NLPHL.

Several studies have demonstrated that the persistent activation of NF-kappaB in HRS cells sustains proliferation and prevents Hodgkin’s lymphoma cells from undergoing apoptosis. Different mechanisms underlie this phenomenon: gains of 2p involving the *REL* locus oncogene (in 30% of the cases), aberrant activation of I-kappaB kinase, mutations of *CYLD*, gene rearrangements and amplification of transcriptional co-activator *BCL3*, and defects of the natural inhibitors of NF-kappaB as the I-kappaB family or as A20, which is encoded by the *TNFAIP3* gene ( 40% of the cases).^44^,^46^–^49^ Additional factors contributing to this activation can be constitutive activated AP-1 as well as CD30 and LMP-1 overexpression.^50^–^52^

Furthermore, HRS cells are characterized by JAK/STAT signalling pathway activation. *JAK2* shows chromosomal gains in about 20% of CHL, and in rare cases is translocated;^53^–^55^ the amplified genomic region on chromosome 9p24, where the *JAK2* gene is located, also includes the gene *JMJD2C* and the programmed death 1 ligand (PD-1L) genes *PD-L1* and *PD-L2* ).^56^–^58^ PD-1Ls can inhibit PD-1–expressing T cells and thereby may contribute to an immunosuppressive microenvironment in CHL.^58^ *JMJD2C* (a histone demethylase) and *JAK2* cooperatively remodel the CHL epigenome and some CHL lines (KM-H2 and L540) are killed when JAK2 and JMJD2C are simultaneously inhibited.^56^ *SOCS1*, a main inhibitor of STAT activity, is affected by inactivating mutations in 40% of CHL cases.^57^

Recently, Tiacci et al.^59^ performed for the first time, a genome-wide transcriptional analysis of microdissected HRS cells from frozen biopsies, compared with other non-Hodgkin B-cell lymphomas (B-NHLs), CHL lines (HDLM2, KMH2, L1236, L428) and normal B-cell subsets. Primary and cultured HRS cells showed similar overall levels of hallmark CHL gene signatures as strong NF-κB activity and downregulation of the B-cell program, although vast transcriptional differences (affecting almost 2000 named genes) were observed. These divergences are probably due to the intimate crosstalk existing between HRS cells and the rich cellular microenvironment, leading to the transcriptional enrichment in primary HRS cells of microenvironment-related processes, such as chemotaxis, cell adhesion, and extracellular matrix remodelling. Moreover the authors demonstrated that EBV infection of HRS cells has a minor transcriptional influence on the established CHL clone. Interestingly, although CHL appears a distinct lymphoma entity overall, HRS cells of its histologic subtypes diverged in their similarity to other related lymphomas: NS-CHL appeared more similar to primary mediastinal B-cell lymphoma cells than MC- and LR-CHL. Conversely, LR-CHL appeared to be very close to NLPHL, suggesting that it has features intermediate between CHL and NLPHL not only in the microenvironment but also in the tumour cells themselves. It is also intriguing that the LR- and MC-CHL subtypes, the closest to NLPHL resulted also the closest to TCRBL, a variant of DLBCL into which NLPHL can transform and whose reactive background can mimic MC-CHL microenvironment. Finally HRS cells displayed deregulated expression of several genes potentially highly relevant to lymphoma pathogenesis, including silencing of the apoptosis-inducer *BIK* and of *INPP5D*, an inhibitor of the PI3K-driven oncogenic pathway.

### Epstein-Barr virus infection

EBV studies reveal infection of neoplastic cells of CHL in a variable percentage of patients depending on the histotypes (Figure 2, l).^18^ In particular, in Western Countries 20–40% of the nodular sclerosis and lymphocyte depleted cases and 50–75% of the mixed cellularity ones reveal expression of LMP1, LMP2a and/or EBER-1/2, but not EBNA2, thus showing a pattern characteristic of latency type II EBV infection. Interestingly enough, these percentages can remarkably vary according to the geographic area examined, i.e. 90% of pediatric case in Africa are EBV positive.^18^ The type of EBV strain also varies between different geographic areas: in developed countries strain 1 prevails, in developing countries strain 2. CHL which is positive for EBV at diagnosis is usually also positive at relapse with persistence of the same EBV strain. In addition, most if not all HIV^+^ HL cases display positivity of neoplastic cells for EBV.^18^ The exact role of EBV in the pathogenesis of classical HL is still open to question. LMP-1 mimics signalling of the active CD40 receptor, an essential co-stimulatory molecule for B-cells, that stimulates NF-kappaB pathway activation.^60^ LMP-2a carries a cytoplasmic motif that resembles the signalling module of the B-cell receptor (BCR).^61^ As CD40 and BCR signalling are the main regulators of survival and selection of B cells in the setting of germinal center (GC) reaction, it was speculated that LMP-1 and LMP-2a can rescue BCR-deficient B cells from apoptosis by replacing these signals.^61^ Indeed, EBV-immortalized B cell lines can be established from BCR-deficient GC B cells.^62^,^63^ Interestingly, the CHL cases carrying crippling mutations of *IG* genes are EBV positive. 64 This suggests that EBV might play a major role as an initial event in HL pathogenesis by rescuing crippled GC B cells from apoptosis. Notably, most *TNFAIP3*-mutated CHLs are EBV negative, indicating that A20 inactivation and EBV infection are largely mutually exclusive transforming events in classical HL.^46^

However, the detection of EBV^+^ HRS-like cells is not a specific finding of CHL. In peripheral T cell lymphoma of the NOS type as well as angioimmunoblastic T cell lymphoma may be encountered EBV^+^ HRS-like of B cell lineage that may simulate CHL. EBV^+^ diffuse large B-cell lymphoma of the elderly, now recognized as a provisional entity in the 4th edition of WHO classification, can show some morphologic overlap with CHL, also encountered in the elderly but reported to have a better prognosis.^65^ Furthermore, Dojcinov et al.^66^ have recently described a new clinicopathologic entity, characterized by EBV^+^ mucocutaneous ulcer with Hodgkin-like features and a self-limited, indolent course, associated with various forms of immunosuppression and generally responding well to conservative management.^66^

## Figures and Tables

**Table 1:** World Health Organisation classification of Hodgkin Lymphoma.

**Nodular lymphocyte predominant HL** **Classical Hodgkin lymphoma** Nodular sclerosis CHL (grades 1 and 2) Mixed cellularity CHL Lymphocyte rich CHL* Lymphocyte depleted CHL

* This includes a nodular (common) and a diffuse (rare) form.

**Table 2:** Classification of NLPHL sec. Fan Z et al ( Am J Surg Pathol 2003).

Classical nodular pattern, B-cell rich (pattern A) Serpiginous/interconnected nodular pattern (pattern B) Nodular, with prominent extra-nodular B-cells (pattern C) Nodular, with T-cell rich background (pattern D) Diffuse (TCRBCL-like) (pattern E) Diffuse, “moth eaten” with B-cell rich background (pattern F)
